# DCGL v2.0: An R Package for Unveiling Differential Regulation from Differential Co-expression

**DOI:** 10.1371/journal.pone.0079729

**Published:** 2013-11-20

**Authors:** Jing Yang, Hui Yu, Bao-Hong Liu, Zhongming Zhao, Lei Liu, Liang-Xiao Ma, Yi-Xue Li, Yuan-Yuan Li

**Affiliations:** 1 School of Biotechnology, East China University of Science and Technology, Shanghai, P. R. China; 2 Bioinformatics Center, Key Laboratory of Systems Biology, Shanghai Institutes for Biological Sciences, Chinese Academy of Sciences, Shanghai, P. R. China; 3 Shanghai Center for Bioinformation Technology, Shanghai Industrial Technology Institute, Shanghai, P. R. China; 4 Department of Biomedical Informatics, Vanderbilt University School of Medicine, Nashville, Tennessee, United States of America; 5 Departments of Psychiatry, Vanderbilt University School of Medicine, Nashville, Tennessee, United States of America; 6 Department of Cancer Biology, Vanderbilt University School of Medicine, Nashville, Tennessee, United States of America; University of California, Los Angeles, United States of America

## Abstract

**Motivation:**

Differential co-expression analysis (DCEA) has emerged in recent years as a novel, systematic investigation into gene expression data. While most DCEA studies or tools focus on the co-expression relationships among genes, some are developing a potentially more promising research domain, differential regulation analysis (DRA). In our previously proposed R package DCGL v1.0, we provided functions to facilitate basic differential co-expression analyses; however, the output from DCGL v1.0 could not be translated into differential regulation mechanisms in a straightforward manner.

**Results:**

To advance from DCEA to DRA, we upgraded the DCGL package from v1.0 to v2.0. A new module named “Differential Regulation Analysis” (DRA) was designed, which consists of three major functions: *DRsort*, *DRplot*, and *DRrank*. *DRsort* selects differentially regulated genes (DRGs) and differentially regulated links (DRLs) according to the transcription factor (TF)-to-target information. *DRrank* prioritizes the TFs in terms of their potential relevance to the phenotype of interest. *DRplot* graphically visualizes differentially co-expressed links (DCLs) and/or TF-to-target links in a network context. In addition to these new modules, we streamlined the codes from v1.0. The evaluation results proved that our differential regulation analysis is able to capture the regulators relevant to the biological subject.

**Conclusions:**

With ample functions to facilitate differential regulation analysis, DCGL v2.0 was upgraded from a DCEA tool to a DRA tool, which may unveil the underlying differential regulation from the observed differential co-expression. DCGL v2.0 can be applied to a wide range of gene expression data in order to systematically identify novel regulators that have not yet been documented as critical.

**Availability:**

DCGL v2.0 package is available at http://cran.r-project.org/web/packages/DCGL/index.html or at our project home page http://lifecenter.sgst.cn/main/en/dcgl.jsp.

## Introduction

In the transcriptome analysis domain, differential co-expression analysis (DCEA) is emerging as a unique complement to traditional differential expression analysis. Rather than calculating expression level changes of individual genes, DCEA investigates differences in gene interconnection by calculating the expression correlation changes of gene pairs between two conditions. In the past few years, a large variety of DCEA methods have been developed, such as Log Ratio of Connectivity (LRC) [1], Average Specific Connectivity (ASC) [2], Weighted Gene Co-expression Network (WGCNA) [3], [4], Differential Co-expression profile (DCp) [5], [6], Differential Co-expression enrichment (DCe) [5], [6], ROS-DET [7], Gene Set Co-expression Analysis [8], and others. These methods vary in how they specify expression correlations and quantify differential co-expression; they also differ in the levels they address: genes or gene sets. As a promising alternative to differential expression analysis, DCEA is drawing increasing attention from computational biologists and, thus, is undergoing rapid methodological improvement.

The rationale behind differential co-expression analysis is that changes in gene co-expression patterns between two contrasting phenotypes (e.g., healthy and disease) provide hints regarding the disrupted regulatory relationships or affected regulatory subnetworks specific to the phenotype of interest (in this case, the disease phenotype). Therefore, among the many growing directions of DCEA, there is the so-called “differential regulation analysis” (DRA), which integrates the transcription factor (TF)-to-target information to probe upstream regulatory events that account for the observed co-expression changes. Recently, researchers have integrated differential co-expression and differential expression concepts to propose a novel Regulatory Impact Factor (RIF) that can be used to prioritize disease-causative TFs [9], [10]. In addition, researchers have begun to perform differential co-expression analyses of microRNAs [11], [12]. These studies are expected to lead to DRA at the post-transcription level. While the algorithm/theory facet of DRA is on the rise, the tool/application facet is lagging. The few existing tools, such as CoXpress [13] and DiffCorr [14], are fine-tuned at the DCEA stage but have not been expanded to the DRA front. Recent DRA methods, such as the RIF metric mentioned above, have not been implemented as practical tools. Currently, a software tool that implements the frontier DRA methods would fill this gap and consequently propagate DRA methods to many more biomedical research fields.

Three years ago, we released the R package DCGL (referred to as DCGL v1.0 hereafter) [6], which was designed to identify differentially co-expressed genes and links (DCGs and DCLs, respectively). The DCGL package facilitated the application of our DCEA algorithms DCp and DCe [5] in a diverse array of disease studies [15]–[18]. In our current work, we introduce an upgraded version of DCGL (referred to as DCGL v2.0 hereafter), in which we added ample functions to facilitate differential regulation analyses. Specifically, we incorporated the human TF-to-target library into the package, achieved the identification of differentially regulated genes and links (DRGs and DRLs, respectively), enabled a network display of intertwined regulation links and differential co-expression links, and implemented the RIF metric as well as two additional novel regulator prioritizing metrics. We have managed to turn the DCGL package into a comprehensive DRA tool. Our case study in hepatocellular carcinoma gene expression studies demonstrated the usage and applicability of DCGL v2.0 in human diseases.

## Methods and Implementations

### Modification of the Existing Modules in DCGL v1.0

**Figure 1:**
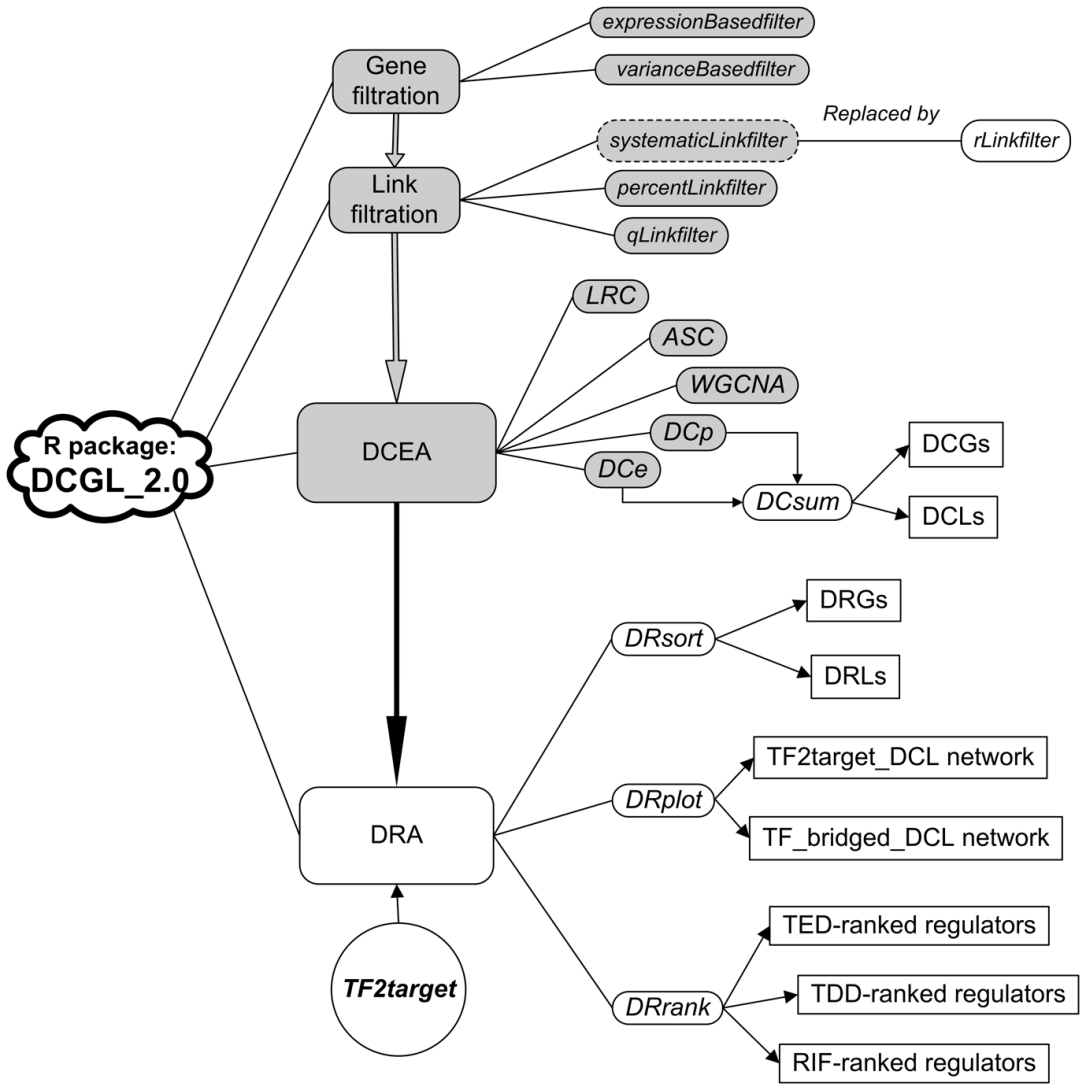
Overall design of DCGL v2.0. Boxes in light grey are modules/functions common to both DCGL v1.0 and v2.0.

As illustrated in Figure 1, the previous DCGL package [6], DCGL v1.0, has three functional modules: Gene filtration, Link filtration, and DCEA (short for differential co-expression analysis). The “Gene filtration” module provide two functions, *expressionBasedfilter* [19] and *varianceBasedfilter* [20], to filter out genes whose expression values are extremely low or notably invariable across samples/conditions. The “Link filtration” module includes three functions, *qLinkfilter*, *percentLinkfilter* and *systematicLinkfilter*, which are designed to construct gene co-expression networks. The “DCEA” module has three algorithms previously proposed by others (*LRC* [1], *ASC* [2], and *WGCNA* [3], [4]) and two methods (*DCp* and *DCe* [5], [6]) we developed to identify differentially co-expressed genes (DCGs) and differentially co-expressed links (DCLs). These existing modules were improved in DCGL v2.0 as follows. 1) The source codes were re-organized to a more logical and efficient level. 2) A stand-alone function, *rLinkfilter*, was added to the “Link filtration” module, which cuts off links according to their expression correlation value. *SystematicLinkfilter,* used to be in DCGL v1.0, was removed from the update because it is extremely time-consuming [21], and its results require manual interpretation before they are applied to downstream functions. 3) A *DCsum* function was attached to module DCEA in order to summarize a final set of DCGs and DCLs (see the companion vignette for more details, Text S1).

### DRA: a New Module in DCGL v2.0

In DCGL v2.0, we designed and implemented a new module, DRA, specifically for differential regulation analysis. The human gene regulatory relationships were developed from the “tfbsConsSites.txt” and “tfbsConsFactors.txt” files extracted from UCSC hg19 (http://genome.ucsc.edu/), and were compiled as the data library *TF2target*. *TF2target* includes 214,607 binary tuples involving 215 human TFs and 16,863 targets (see the companion vignette for more details, Text S1). In order to keep abreast of developments in human regulatory data analysis, we will continue to tidy and promote our *TF2target* library. The DRA module is comprised of three major functions, *DRsort, DRplot,* and *DRrank*. Briefly, *DRsort* sorts differentially regulated genes (DRGs) and differentially regulated links (DRLs) from the *DCsum*-outputted DCGs and DCLs. *DRplot* visualizes the networks of intertwined regulation links and DCLs. *DRrank* prioritizes candidate causal regulators using three alternative metrics.

### *DRsort*: Sorting Out Potential DRGs & DRLs

**Figure 2:**
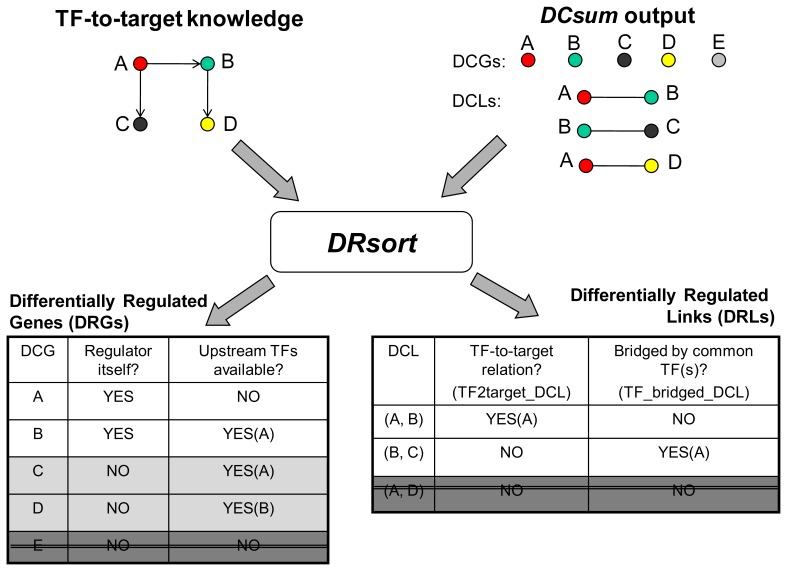
*DRsort* functionality: sifting the DRGs and DRLs with regulation knowledge. Given the TF-to-target knowledge (top left) as a reference, *DRsort* highlights a subset of differentially co-expressed genes and links (top right) as either differentially regulated genes (bottom left) or differentially regulated links (bottom right). As they are sorted, some DCGs/DCLs are discarded (dark grey rows with double strikethrough), while some DCGs, though they are not termed DRGs themselves, are reserved to ease downstream analysis (light grey rows in the bottom left table).

As the foremost function of the DRA module, *DRsort* is designed to sift differentially regulated genes (DRGs) and differentially regulated links (DRLs) from the *DCsum*-outputted DCGs and DCLs. In this function, we scrutinize the DCGs and DCLs against the TF-to-target information and highlight a subset of the genes and links that are potentially highly related to the putative differential regulation mechanisms. If a DCG coincides with a TF (A and B in the left table in Figure 2), it is regarded as a differentially regulated gene (DRG, or TF DCG) based on the implication that a differential co-expression of this type of DCGs could be attributed to disrupted regulatory relationships between the TF and its targets. If a DCG is not a TF by itself, but its regulator(s) is/are traceable in *TF2target* (C and D in the left table in Figure 2), this DCG, though not regarded as a DRG, is reserved in a *DRsort* output to ease downstream analyses.

Among all DCLs, we obtained two types of DRLs, namely TF2target_DCLs and TF_bridged_DCLs. “TF2target_DCLs” refers to DCLs that coincide with TF-to-target relations (for example, the edge between A and B in the DRL table in Figure 2), while “TF_bridged_DCLs” refers to DCLs for which both genes share common TF(s) ((B, C) in the right table in Figure 2). Our rationale here is that the disruption of regulatory relations can affect not only the co-expression links between a regulator and its targets (TF2target_DCL), but also the co-expression links among the multiple targets of a TF (TF_bridged_DCL).

### *DRplot*: Visualizing Differential Co-expression and Regulatory Relationships

**Figure 3:**
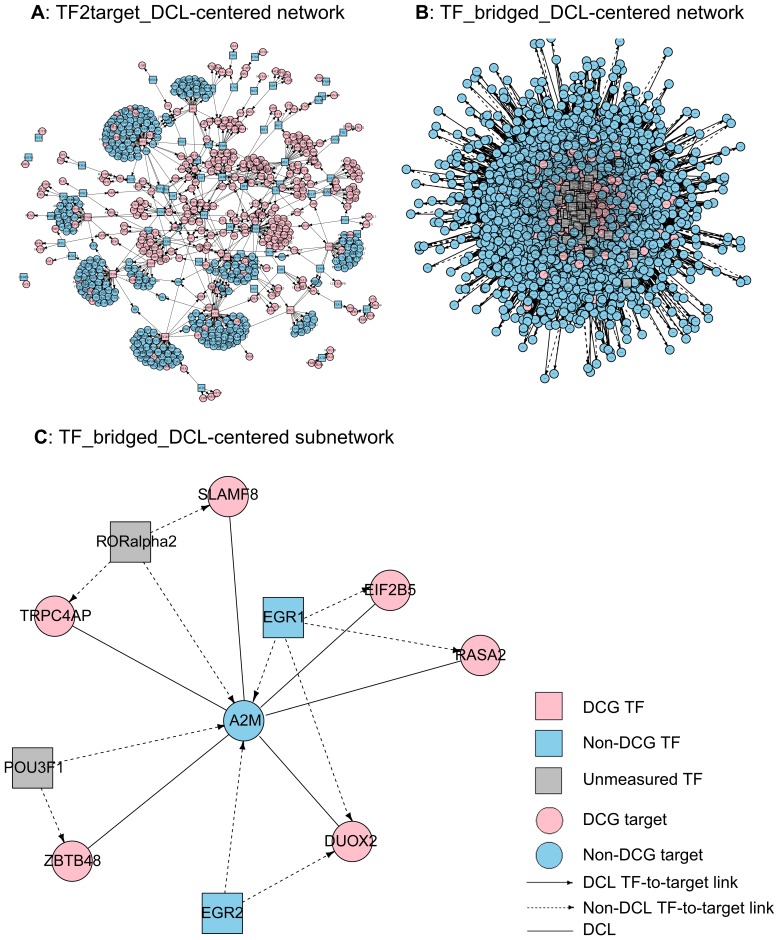
Example DRL-centered heterogeneous networks produced using the *DRplot* function. GSE17967 was used as the sample dataset. Nodes denote genes, and edges denote DCLs or TF-to-target links (see symbol illustration). A, TF2target_DCL-centered network contains 663 genes and 751 links. B, TF_bridged_DCL-centered network contains 6,207 genes and 294,117 links. C, A subnetwork out of the TF_bridged_DCL-centered network surrounding the predefined gene *A2M*.

Given the DRGs’ and DRLs’ output from *DRsort* as well as the TF-to-target regulatory relationships in *TF2target*, we developed a *DRplot* function to visualize a DRG-highlighted, DRL-centered network. By definition, our DRLs involved differential co-expression links and transcriptional regulation links, consequently leading to a heterogeneous network display. We offer two network plotting options to present the two types of DRLs separately: TF2target_DCLs (Figure 3A) and TF_bridged_DCLs (Figure 3B). In addition, we allow users to delimit a sub-network according to predefined gene(s) of interest, where the predefined genes involving DRLs and regulation links are extracted from the whole network (Figure 3C).

We utilized dataset GSE17967 [22] from GEO (http://www.ncbi.nlm.nih.gov/geo/) to demonstrate the function of *DRplot*. This dataset was also used in the subsequent steps for *DRrank* illustration. The details of data processing and analyses can be found in the section “Results: Assessment of DCGL v2.0.”

### *DRrank*: Ranking Regulators

Finally, in *DRrank,* we implemented three alternative metrics for prioritizing regulators that are putatively causative to the phenotype of interest. The TED and TDD scores, short for “Targets’ Enrichment Density” and “Targets’ DCL Density,” respectively, are novel inventions in light of our *DRsort* analysis. In addition, the “Regulatory Impact Factor” (RIF score) established earlier [9], [10] was also implemented in our package.

Similar to the inferences made in previous works regarding the relationship between TFs and differentially expressed genes (DEGs) [23]–[25], we speculate that a TF must be more subject-relevant or even causative if more of its targets are DCGs. Based on this speculation, TED evaluates the enrichment of a particular TF’s targets in DCGs using the binomial probability model. While an overall set of *K* DCGs are determined from a total of *N* genes with available expression data, out of which N_0_ genes (K_0_ DCGs) are covered by *TF2target* library, a TF (*TF_i_*) with *Ti* targets out of *N*_0_ genes should by chance have Ti*K_0_ */N*_0_ targets fall within the *K*_0_ DCGs. If the actual number of its DCG targets, *Ti’*, is significantly larger than the expected number, Ti*K_0_ */N*_0_, as judged by the cumulative density function of the binomial probability model (Eq. 1), we tend to rank *TF_i_* higher in the regulator prioritization list. That is, if more DCGs are enriched in the targets, then the regulator is more prioritized. Of note, TED is applicable to any TF as long as the expression level of its targets are measured, which means its own expression information is not required.
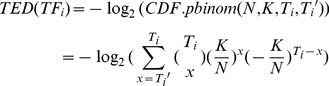
(1)


Still using GSE17967 as an example, this dataset tested a total of 12,632 genes, out of which 1,052 genes were identified as DCGs. Taking the simplified scenario of 13 genes and 23 links in Figure 3C as an example, the TF *EGR1* (*Egr-1*) has four regulatory targets covered by GSE17967. By chance, *EGR1* should have 4*1,052/12,632 DCG targets; however, *EGR1*’s real number is three. According to Eq. 1, we have *TED(EGR1)* = 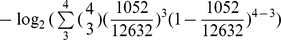 = 14.34351.

TDD is designed to prioritize TFs whose targets form DCLs (i.e., “common TFs” of the TF_bridged_DCLs). Bearing the same heuristic approach as in TED, we speculate that a TF of higher importance should have more of its targets forming DCLs. Based on this speculation, we borrowed the “clustering coefficient” formula [26] to measure the relevance of a TF(Eq. 2). For TF*_i_*, if we identify *N* targets that have available expression data, among which *k* DCLs are formed, then TDD is essentially a normalized number of TF_i_-bridged DCLs (Eq. 2). As with TED, TDD can rank those TFs that are not tested in an expression dataset as long as their targets’ expression information is available.
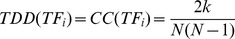
(2)


Again, based on GSE17967, *EGR1* has four targets with expression data, among which three DCLs are formed (Figure 3C). According to Eq. 2, we have *TDD(EGR1)* = 2*3/4(4–1) = 0.5.

The regulatory impact factor (RIF) was recently proposed and demonstrated as efficient in a proof-of-concept study of bovine Piedmontese myostatin mutants [9], [10]. The RIF measurement simultaneously integrates three sources of information: (i) the extent of differential expression; (ii) the abundance of differentially expressed genes; and, (iii) the differential co-expression between a TF and its differentially expressed target genes (Eq. 3). In other words, the RIF algorithm assigns a high score to those TFs that are “cumulatively most differentially wired to the abundant most differentially expressed genes” [9]. In combination, these factors are assumed to contribute to the relevance of a TF in relation to the phenotype under research.
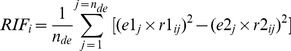
(3)


In Eq. 3, *n_de_* is the number of the differentially expressed gene (DEG); *e1_j_* or *e2_j_* denotes the expression value of DEG_j_ in an experimental condition (1 or 2); *r1_ij_* or *r2_ij_* designates the correlation between TF_i_ and DEG_j_ [27].

To evaluate the statistical significance of TED and TDD scores, we implement a permutation test to provide p-values as well as false discovery rate (FDR) values in conjunction with the TED/TDD scores. We randomly designate an unchanged number of pseudo targets to each TF and calculate a pseudo TED or TDD score. The number of repeated permutations can be chosen by the user (0 by default). A large number (e.g., 1,000) of pseudo TED or TDD statistics form an empirical null distribution from which the p-value can be estimated and FDR value can be obtained accordingly.

## Results: Assessment of DCGL v2.0

### Validation of Differential Regulation Analysis Methods

We utilized dataset GSE17967 [22] to demonstrate the utility of the new functions in DCGL v2.0. GSE17967 was designed to detect gene expression in cirrhotic tissues with (sample number = 16) and without (sample number = 47) hepatocellular carcinoma (HCC). First, a total of 1,052 DCGs and 787,150 DCLs were summarized by *DCsum* based on *DCp* and *DCe* results. The 787,150 DCLs involved 7,533 genes. These DCGs and DCLs were then used as inputs for the differential regulation analysis (DRA) pipeline, and we obtained the following major results.

*DRsort* identified 10 DRGs, 751 TF2target_DCLs (*i.e.* Type I DRLs), and 215,897 TF_bridged_DCLs (*i.e.* Type II DRLs). The total 216, 648 DRLs involved 6,068 genes. We found that *DRsort* here achieved a significant enrichment of the human cancer-related genes (obtained from “Cancer Gene Census,” http://www.sanger.ac.uk/genetics/CGP/Census/) when it sifted DRGs/DRLs from DCGs/DCLs (Table 1).

**Table 1:** Numbers of *DCsum*/*DRsort* result items and the enrichment of cancer genes from *DCsum* result to *DRsort* result.

Comparison	Result items	Total Number	Cancer Gene Total	Cancer Gene Enrichment*
From DCGs to DRGs	DCGs	1,052	27	1.2×10^−4^
	DRGs	10	3	
From DCLs to DRLs	Genes in DCLs	7,533	216	7.1×10^−4^
	Genes in DRLs	6,068	191	

* Cancer Gene enrichment is shown as a p-value resulting from a binomial probability model using the four total numbers in the left columns.

*DRplot* plotted two types of networks, TF2target_DCL-centered (Figure 3A) and TF_bridged_DCL-centered (Figure 3B). A sub-network of the TF_bridged_DCL-centered network is displayed in Figure 3C, which was determined using an HCC relevant gene, *A2M* [28].

Of the total 215 TFs included in the *TF2target* library, 131 had expression data available in our expression dataset. As a consequence, TED and TDD produced rankings of all 215 TFs, while RIF gave its ranking of the 131 TFs with available expression data.

According to the heuristic approach underlying DCEA, it is assumed that the following TFs are more likely to be implicated in the putative differential regulation mechanisms: Type I TFs, i.e. TFs that are DCGs by themselves (DRG, or TF DCGs); Type II TFs, i.e. TFs that change co-expression links with their targets (regulators involved in TF2target_DCLs); and Type III TFs, i.e. TFs whose targets form DCLs (“common TF” involved in TF_bridged_DCLs). In this case, we found 10 Type I TFs, 72 Type II TFs, and 215 Type III TFs. The 10 Type I TFs were *MYC, EP300, LMO2, FOXO1, EGR1, ZIC1, NR3C1, FOSB, GCGR,* and *STAT6*. Of them, *MYC*, *EP300,* and *LMO2* are annotated as cancer genes in “Cancer Gene Census” (http://www.sanger.ac.uk/genetics/CGP/Census/). Literature mining informed us that *FOXO1* [29] and *EGR1* [30] have been implicated in HCC, and *ZIC1* is down-regulated in gastric cancer [31] and has been proved to be a tumor suppressor gene in colorectal cancer [32], [33]. *NR3C1* is identified as an epigenetically deregulated gene in colorectal tumorigenesis [34]. As for the 72 Type II TFs, they were shown as significantly enriched for “KEGG_PATHWAY:hsa05200:pathways in cancer” by DAVID [35] (FDR = 4.76 × 10^−7^) (see Text S3 for KEGG enrichment analysis result of Type II TFs). The high bias of Type I and Type II TFs towards cancer genes in this case study supported our heuristic assumption regarding these particular TFs. Since Type III TFs spanned all 215 TFs included in *TF2target*, they were ignored in the functional enrichment analysis.

Next, we investigated the ranks of the above plausibly relevant TFs in the prioritization lists by TED, TDD, and RIF, respectively (see Text S2 for *DRrank* results for Type I, II and III TFs). It was found that Type I TFs and Type II TFs were significantly highly ranked in the 215-gene list when utilizing TED and TDD, yet this was not the case using RIF (column “Type I TF” and “Type II TF” in Table 2). Since Type III TFs cover all 215 TFs, it is impossible to carry out a comparative evaluation of the three regulator-ranking metrics based on them.

**Table 2:** Wilcox test p-values of particular genes’ top-ranking in alternative regulator prioritization lists.

Prioritization Metrics	Type I TFs (10)	Type II TFs (72)	Type III TFs (215)^a^	Cancer Genes (27)
TED	0.026*	0.004*	–	0.007*
TDD	0.041*	0.040*	–	0.006*
RIF	0.076	0.375	–	0.489

Investigated was the positioning of four types of important genes in three Prioritization lists (by metrics TED, TDD, and RIF). Numbers of considered genes are shown in brackets.

* Statistical significance (p<0.05).

a Since Type III TFs coincide with all TFs, they are ignored in this analysis.

We then extracted the 27 cancer genes from the 215 TFs and discovered that these 27 genes were also significantly highly ranked in the 215-gene list when utilizing TED and TDD, yet this was not the case using RIF (column “Cancer Genes” in Table 2). These observations establish the validity of the TED and TDD designs.

### Evaluation of Computational Efficiency Promotion

In DCGL v2.0, the source codes of pre-existing functions were refined/re-organized into a more logical and efficient form. We tested to see if the coding optimization enhanced computational efficiency. For the convenience of backward comparison, dataset GSE3068 [36], adopted as the benchmark dataset in DCGL v1.0, was utilized to demonstrate the promoted computational efficiency. We performed a series of numerical experiments over varied subsets of GSE3068 using the shared functions from DCGL v1.0 and v2.0, respectively. The computation time used by DCGL v2.0 functions was significantly reduced (Table 3).

**Table 3:** Computing time of shared functions implemented in DCGL v1.0 and DCGL v2.0, tested on different subsets of gene expression datasetGSE3068.

	Number of genes						
Function	1,000	2,000	3,000	4,000	5,000	6,000	7,000
DCp.percent.v1.0	0.27	1.35	2.50	4.05	5.23	8.62	12.02
DCp.percent.v2.0	0.27	0.79	1.79	3.83	4.97	7.29	10.29
DCp.qth.v1.0	0.40	2.06	3.78	6.55	8.74	13.78	19.89
DCp.qth.v2.0	0.38	1.32	3.66	5.89	7.72	13.33	19.28
DCe.percent.v1.0	0.54	2.45	4.81	9.65	13.40	18.27	25.10
DCe.percent.v2.0	0.29	1.42	4.34	6.73	9.62	15.90	18.13
DCe.qth.v1.0	0.46	1.46	3.74	6.73	11.95	15.27	25.93
DCe.qth.v2.0	0.12	1.03	3.11	5.67	9.62	11.22	16.67

Different subsets, with a gradually increasing number of genes, were taken from GSE3068 by selecting the upper rows of the full dataset. The computing platform was a Linux system with five nodes, each of which had a dual quad-core Intel Xeon 2.33GHZ CPU and a RAM of 16 GB. Execution time was averaged over three repetitive runs each.

## Discussion

Identifying the regulators that are relevant or even causative to a phenotypic change is a challenging and worthwhile goal for both experimental and computational biologists. Unfortunately, this problem cannot be solved using differential expression analysis alone. The first reason for this limitation is that causal signals are always submerged within a large amount of differentially expressed genes. More importantly, however, a causal regulator is not necessarily differentially expressed. For example, if a mutation occurs to the activation domain of a TF, the TF, while at its original expression level, can no longer activate its target genes. Another similar case is the regulation of a TF at the post-translational level, which can hamper the TF’s functionality but not its expression level. In either a TF’s missense mutation or its post-translational modification, the expression correlation between the TF and its targets can be affected; this phenomenon might be captured using the Differential Co-Expression Analysis (DCEA) and Differential Regulation Analysis (DRA) methodologies [1]–[7]. Indeed, a differential wiring analysis of expression data succeeded in identifying the gene containing the causal mutation in bovine Piedmontese myostatin mutants [9]. Although we cannot routinely identify those causal regulators at the current stage, differential co-expression analysis has gained wide acknowledgement as a promising method to solve this problem [37]. From a practical viewpoint, developing efficient differential regulation analysis methods and implementing the currently available algorithms is crucial.

The present package DCGL v2.0, upgraded from the earlier version DCGL v1.0 [6], has realized a differential co-expression analysis and, furthermore, a differential regulation analysis pipeline. This upgrade enabled the identification of DCGs and DCLs, the scrutinization of DRGs and DRLs, and, more importantly, the prioritization of potential causal regulators in terms of their relevance or causativeness to a specific phenotype. We have implemented not only the recently proposed RIF method [9], but also two other self-proposed novel ones, TED and TDD. Last, but not least, we created a user-friendly graphic view of the differential co-expression/differential regulation networks. To the best of our knowledge, DCGL v2.0 is the first R package that provides convenient and practical DRA functionalities.

The prioritization of candidate regulators, or the identification of critical regulators, is the toughest and most intriguing part of differential regulation analysis. If one can properly integrate the expression information and regulatory knowledge in a biologically relevant manner, there will be a greater chance to identify true causal factors. Taking the RIF measure as an example [10], by combining the extent of differential expression, the abundance of differentially expressed genes, and differential co-expression between TFs and differentially expressed targets, this approach could capture those regulators that are cumulatively most differentially wired to the abundant most differentially expressed genes. As an efficiency-validated metric, RIF is implemented in DCGL for users’ convenient utilization.

In our design of DRG/DRL selection and regulator prioritization approaches, we also aimed to make full use of the expression information and regulatory knowledge available between TFs and targets. On one hand, the plausibly relevant TFs, TF DCGs, TFs in DCLs, and TFs shared by DCL gene pairs were catalogued in our *DRsort* output for potential intensive examination, as demonstrated in our hepatocellular carcinoma case study. On the other hand, when prioritizing the candidate regulators, TED and TDD examine different aspects of differential regulation. TED assigns a high score to those regulators that regulate more DCGs, while TDD attributes a high score to those whose targets form more DCLs. In our case study, our novel metrics TED and TDD seemed to outperform RIF since they were better at prioritizing phenotype (cancer) related genes. Additionally, our two novel metrics are unique in that they can work on any TFs as long as their target genes’ expression information is available. In contrast, RIF requires the expression information of the regulator itself. A systematic comparative evaluation of the regulator-prioritization metrics remains for future continuous study. However, we decided to implement all of these three measures in DCGL v2.0 since different approaches identify different sets of genes that may contribute to different parts of the process of interest.

In conclusion, DCGL v2.0 implements valuable differential co-expression analysis and differential regulation analysis methodologies. It has universal applicability and is suitable for both microarray data and RNA-seq data. With the present update, DCGL could be used to systematically identify novel TFs contributing to phenotypic change that have not yet been documented as critical, thereby significantly increasing the biological knowledge that could be derived from expression data.

## Availability and Requirements

Project name: DCGL_2.0.

Project stable release: http://cran.r-project.org/web/packages/DCGL/index.html.

Project home page: http://lifecenter.sgst.cn/main/en/dcgl.jsp.

Operating system(s): Platform Independent.

Programming language(s): R.

Other requirement(s): R, R packages (igraph, limma).

License: GPL (>2).

## Supporting Information

Text S1 **DCGL v2.0 Vignette.**
(PDF)

Text S2 ***DRrank*** **result for the GSE17967 dataset.**
(TXT)

Text S3 **KEGG enrichment analysis result of Type II TFs for the GSE17967 dataset.**
(TXT)
