# Supplementary material for: DCGL v2.0: An R Package for Unveiling Differential Regulation from Differential Co-expression
Source: PLoS One. 2013 Nov 20;8(11):e79729. doi: 10.1371/journal.pone.0079729 (PMC3835854; doi:10.1371/journal.pone.0079729)
Supplement: Text S1 — DCGL v2.0 Vignette. (PDF) [file pone.0079729.s001.pdf]

Using the DCGL_2.0 Package

Jing Yang[1,2], Hui Yu[2] and Bao-Hong Liu[2]

August 12, 2013

[1]School of Biotechnology, East China University of Science and Technology. Shanghai 200237, P.R. China.
[2]Shanghai Center for Bioinformation Technology. Shanghai 201203, P.R. China.

`yangjing@scbit.org and bhliu@scbit.org`

Contents

1 Introduction **2**

2 Getting started **2**

3 Methods **4**
3.1 Gene filtration 4
3.2 Link filtration 4
3.2.1 Filtering gene links according to the correlation threshold 4
3.2.2 Filtering gene links according to the max correlation value 5
3.2.3 Filtering gene links according to the q-values of correlation values . 5
3.3 Differential co-expression analysis 5
3.3.1 DCp for identifying DCGs 5
3.3.2 DCe for identifying DCGs and DCLs 6
3.3.3 WGCNA, ASC and LRC for identifying DCGs 6
3.3.4 DCsum for summarizing DCGs and DCLs 6
3.4 Differential regulation analysis 6
3.4.1 DRsort for sorting out DRGs and DRLs 6
3.4.2 DRplot for visualizing differential co-expression and regulatory relationships 7
3.4.3 DRrank for ranking regulators 7

4 Dataset **9**

5 Examples **9**
5.1 Gene filtration 9
5.2 DCp: Identifying DCGs 10
5.3 DCe: Identifying DCGs and DCLs 11

5.4 DCsum: Summarizing DCGs and DCLs 12
5.5 DRsort: Sorting out DRGs and DRLs 13
5.6 DRplot: Visualizing differential co-expression and regulatory relationships . 14
5.7 DRrank: Ranking regulators 15

6 List of abbreviations used **19**

1 Introduction

This document gives instructions on how to use the functions of *DCGL_2.0* which is an advanced and upgraded version of *DCGL_1.0*. *DCGL_2.0* contains four modules which are Gene filtration module, Link filtration module, differential co-expression analysis (DCEA) module and differential regulation analysis (DRA) module.

In Gene filtration module, there are `expressionBasedfilter` and `varianceBasedfilter` functions to filter genes on expression microarray data. `rLinkfilter`, `percentLinkfilter` and `qLinkfilter` functions were wrapped in Link filtration module to filter gene co-expression links in co-expression networks. `DCp`, `DCe`, `WGCNA`, `LRC` and `ASC` functions were implemented in DCEA module for extracting differentially coexpressed genes (DCGs) and differentially coexpressed links (DCLs). These above functions have been accomplished into *DCGL_1.0*.

In *DCGL_2.0*, we attached to DCEA module a new function, `DCsum`, to determine a final set of DCGs and DCLs which come from multiple DCEA methods. Most importantly, we produced DRA module which contains `DRsort`, `DRplot` and `DRrank` for differential regulation analysis. `DRsort` identifies differentially regulated genes (DRGs) and differentially regulated links (DRLs) from `DCsum`-outputted DCGs and DCLs based on TF-to-target knowledge. `DRplot` visualizes DRLs and DRLs-related TF-to-target links. Function of prioritizing regulators in terms of their potential relevance to the biological phenotype was designed in `DRrank`. Figure 1 shows the overall design of *DCGL_2.0*.

The major input of *DCGL_2.0* are two expression data matrices from two contrastive conditions, where the rows and columns correspond to genes and microarrays respectively. TF-to-target regulation knowledge, which was wrapped in the package, is another required input dataset.

The *DCGL_2.0* package employs R library *igraph*, *limma*, *org.Hs.eg.db*, which must be installed in advance.

2 Getting started

Prior to using *DCGL_2.0*, users should download the installation file of *DCGL_2.0* to their local computer, and install *DCGL_2.0* as a package of their R computing environment. For Linux users, they should type 'R CMD INSTALL DCGL_2.0.tar.gz' in the shell (suppose the installation file 'DCGL_2.0.tar.gz' is in the current working directory); for windows users, they should go to the R menu 'Packages' and click the 'Install package(s) from local zip files' and then locate the local file 'DCGL_2.0.zip'. If the package is installed successfully, a file folder named 'DCGL' should appear beneath the folder 'library' in the R installation directory.

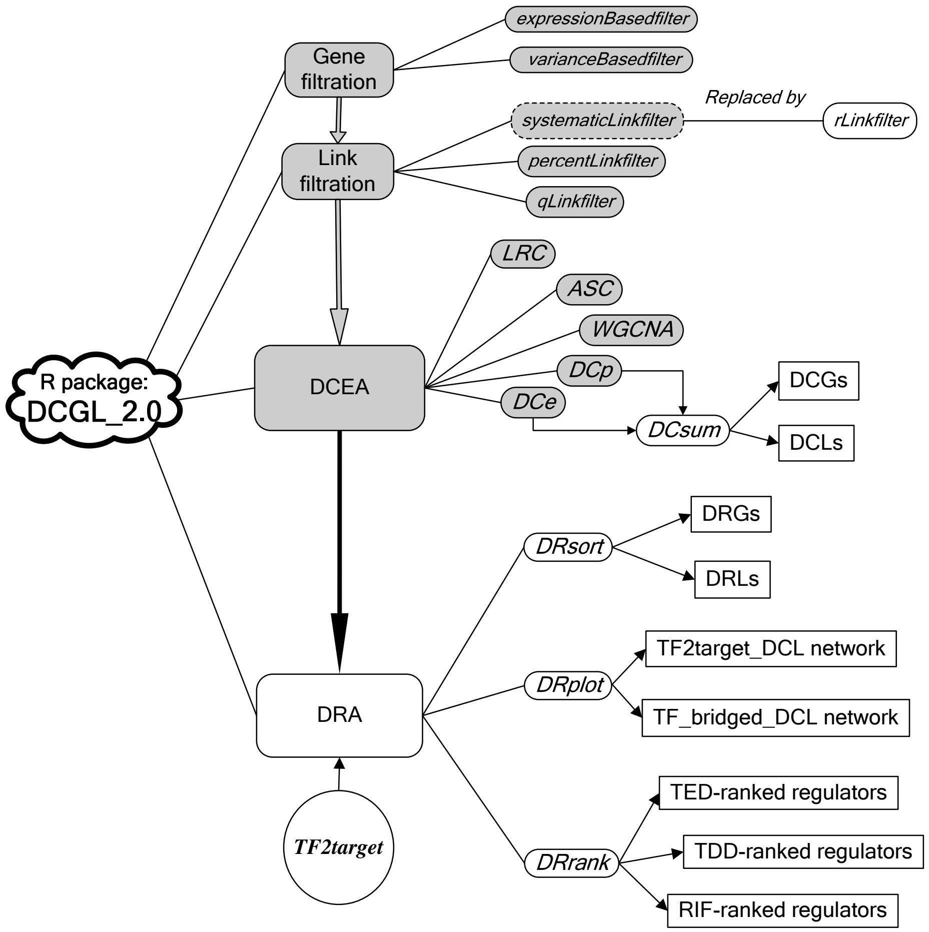

Figure 1: Overall design of DCGL_2.0. Functions implemented in both DCGL_1.0 and D-CGL_2.0 are represented in light gray background. DCEA: differential co-expression analysis; DRA: differential regulation analysis.

To load the *DCGL_2.0* package, type `library(DCGL)`.

3 Methods

DCGL_2.0 provides the pre-existing facilities for gene filtering, link filtering and D-CGs/DCLs identification of *DCGL_1.0*, as well as newly added functions for DCGs/DCLs summarization, DRGs/DRLs identification, networks visualization, and regulators ranking.

3.1 Gene filtration

If there are too many genes in the expression dataset, one can filter out some genes using the `expressionBasedfilter` or `varianceBasedfilter` or both of them. `expressionBasedfilter` filters out a half genes that have their Between-Experiment Mean Expression Signal (BEMES) lower than the median BEMES of all genes (Prieto and etal.,2008). `varianceBasedfilter` is an approximate test of the hypothesis that gene has the same variance as the median variance (Simon and Lam,2006). The variance of the log-values for each gene is compared to the median of all the variances.The *quantity*

$$quantity = (n-1) * var_i / var_m$$

for each gene is compared to a percentile of a chi-square distribution (with a degree of freedom of $n-1$, n being the number of arrays) to filter out those genes not significantly more variable than the median gene.

3.2 Link filtration

For all DCEA methods but WGCNA, a link filtering step is necessary to build up two gene co-expression networks for the two contrastive conditions. The two gene co-expression networks have identical linking structures but different edge weights (co-expression values). The input to link filtering methods always includes two separate gene expression matrices for the two conditions, and the output mainly comprises two data vectors, each coming from a half of the symmetrical gene-versus-gene co-expression matrices. One can imagine that, in the intermediate co-expression matrices, retained links have non-zero values while discarded links are denoted with zero values.

Three stand-alone functions are implemented for link filtering, which are the correlation value threshold (`rLinkfilter`), the correlation-value fraction based link filtering (`percentLinkfilter`) and the q-value based link filtering (`qLinkfilter`). However, these link filtering functions are seldom called as independent functions; instead, they are wrapped in the DCEA functions `DCp`, `DCe`, `ASC` and `LRC`, and can be tuned with the 'link.method' and 'cutoff' parameters.

3.2.1 Filtering gene links according to the correlation threshold

As an argument to the 'link.method' parameter, `rLinkfilter` is abbreviated to 'rth'. Each gene link is associated with two correlation values (one out of condition A and the other out of condition B); if either of the two correlation values is greater than the given correlation threshold ('cutoff'), the gene link is retained.

3.2.2 Filtering gene links according to the max correlation value

As an argument to the 'link.method' parameter, `percentLinkfilter` is abbreviated to 'percent'. Each gene link is associated with two correlation values (one out of condition A and the other out of condition B) and thus a vector of 'maximum absolute values' for all correlation value pairs is decided. Then these 'maximum absolute values' are sorted in decreasing order. At last, a fraction ('cutoff') of gene pairs with the highest max correlation values will be retained.

3.2.3 Filtering gene links according to the q-values of correlation values

As an argument to the 'link.method' parameter, `qLinkfilter` is abbreviated to 'qth'. For each of the two experimental conditions, the co-expression values are associated with the corresponding p-values (student T-test of the zero nature of a Pearson Correlation Coefficient (PCC)), and these p-values are sorted and transformed to q-values (false discovery rates). In this way, each gene link is associated with a pair of q-value, and those links with at least one q-value lower than the threshold ('cutoff') are retained.

3.3 Differential co-expression analysis

DCEA module contains five DCEA methods. `DCp` and `DCe`(Yu and etal.,2011)(Liu and etal.,2010) proposed by us, and `WGCNA`, `ASC`, and `LRC` were proposed by other inventors. All the methods are aimed to extract DCGs/DCLs through analysing the changes of the connections. All methods must be preceded by a link filtering step, which can be tuned with the 'link.method' and 'cutoff' parameters. After the link filtering, co-expression pairs with rth/percent/qth of co-expression values in either of two conditions higher/higher/lower than the cutoff are retained.

3.3.1 DCp for identifying DCGs

DCp works on the filtered set of gene co-expression value pairs, where each pair is made up with two co-expression values calculated under two different conditions separately. The subset of co-expression value pairs associated with a particular gene, in two groups for the two conditions separately, can be written as two vectors X and Y (n is co-expression neighbors for a gene).

$$X = (x_{i1}, x_{i2}, ..., x_{in})$$

$$Y = (y_{i1}, y_{i2}, ..., y_{in})$$

Then a length-normalized Euclidean distance is used for measuring differential co-expression (dC) of this gene.

$$dC_n(i) = \sqrt{\frac{(x_{i1} - y_{i1})^2 + (x_{i2} - y_{i2})^2 + ... + (x_{in} - y_{in})^2}{n}}$$

To evaluate whether a gene has significant dC, we perform a permutation test, in which we randomly permute the disease and normal conditions of the samples, calculate new PCCs, filter gene pairs based on the new PCCs, and calculate new dC statistics. The

sample permutation is repeated N times, and a large number of permutation dC statistics form an empirical null distribution. The p-value for each gene can then be estimated.

3.3.2 DCe for identifying DCGs and DCLs

`DCe` is based on the 'Limit Fold Change' (LFC) model, a robust statistical method originally proposed for selecting differentially expressed genes(DEGs) from microarray data (Mutch and etal.,2002).

First, the correlation pairs are divided into three parts according to the pairing of signs of co-expression values and the multitude of co-expression values: pairs with same signs (N_1), pairs with different signs (N_2) and pairs with differently-signed high co-expression values (N_3). The "high co-expression values" are deemed based on the same correlation value threshold as in the `qLinkfilter` function. The first two parts are processed with the 'LFC' model separately to yield two subsets of DCLs (K_1,K_2), while the third part (N_3) adds to the set of DCLs directly. So a total of K=N_3+K_1+K_2 DCLs are determined from a total of N gene links. For a gene (g_i), the total number of links (n_i) and DCLs in particular(k_i) associated with it are counted, and the Binomial Probability model is used to estimate the significance of the gene being a DCG.

$$P(g_i) = \sum_{x=k_i}^{n_i} C_{n_i}^{x} (\frac{K}{N})^x (1 - \frac{K}{N})^{n_i - x}$$

3.3.3 WGCNA, ASC and LRC for identifying DCGs

`WGCNA` (Fuller and etal.,2007; van Nas and etal.,2009), `ASC` (Choi and etal.,2005) and `LRC` (Reverter and etal.,2005) are other methods for measuring genes' differential co-expression. For more details please consult (Yu and etal.,2011; Liu and etal.,2010) (i.e. *DCGL_1.0*).

3.3.4 DCsum for summarizing DCGs and DCLs

`DCsum`, short for differentially co-expression summarization, summarizes 1) a set of DCGs, which is an intersection of DCp-derived DCGs (selected with a q value cutoff or a given percentage of dC) and DCe-derived DCGs (selected with a q value cutoff), 2) a set of DCLs, which is sifted from DCe-derived DCLs that are connected to at least one DCG determined by the first step. As a result, `DCsum` combines results from two different co-expression analysis methods.

3.4 Differential regulation analysis

3.4.1 DRsort for sorting out DRGs and DRLs

`DRsort`, the first function of DRA module, is aimed to sift DCGs and DCLs according to regulation knowledge (i.e. TF-to-target) which will be introduced in the section of 'Dataset'.

If a DCG is a TF, it is intuitively speculated that its related differential co-expression may be attributed to the change of its regulation relationships with its targets. So this type of DCGs are termed differential regulation genes (DRGs). Besides if the upstream TFs of

a DCG is identified, that DCG is possibly a differentially regulated target of an implicated regulator, and so such DCGs are also kept in the set of DRGs.

If a DCL happens to be a TF-to-target relation, we highlight this DCL because it is the direct attribution to differential regulation. This type of DCLs are termed 'TF2target_DCL'. On the other hand, if there are one or more common TFs regulating the two genes of a DCL, we also give priority to this DCL because the change in the expression correlation of the two genes could be attributed to the disruption of their co-regulation by the common TFs. This type of DCLs are termed 'TF_bridged_DCLs'. TF2target_DCLs and TF_bridged_DCLs, therefore, together form the set of differentially regulated links(DRLs).

3.4.2 DRplot for visualizing differential co-expression and regulatory relationships

We built a function `DRplot` to display combined information of DCGs/DCLs, DRGs/DRLs and TF-to-target. `DRplot` generates DRL-centered networks. Due to the definite of DRL, TFs, TFs' regulation links and DCGs were involved to form two heterogeneous networks which are 1): TF2target_DCL-centered network (Figure 2) and 2): TF_bridged_DCL-centered network (Figure 3). In both networks, we rely on different node shapes to differentiate TFs and non-TFs (square for TFs, circle for non-TFs), different node colors to categorize genes (pink for DCGs, blue for non-DCGs, gray for TFs which are not tested in expression microarray data and therefore cannot be determined as DCGs or not), and different edge types to express different relations of gene pairs (solid for DCLs, dashed for non-DCLs; edges with arrow indicate TF-to-target relations).

In addition, `DRplot` allows user to delimit a sub-network around a predefined set of genes of interest (Figure 4 as an example of TF_bridged_DCL-centered sub-network). DRLs in TF2target_DCL-centered sub-network were extracted from whole TF2target_DCLs when interested gene(s) was/were either gene of a TF2target_DCL. In TF_bridged_DCL-centered sub-network, DRLs were kept when predefined gene(s) was/were either gene of a TF_bridged_DCL or the common TF. Meanwhile corresponding regulation links which regulated by common TF were also extracted.

3.4.3 DRrank for ranking regulators

`DRrank` is implemented for ranking potential TFs in terms of their relevance to the phenotypic change or biophysical process of interest. It contains three methods: RIF (Reverter and etal.,2010), TED, and TDD. The latter two methods were proposed by us firstly in this package.

TED, short for 'Target Enrichment Density', employs Binomial Probability model to quantify the enrichment of a TF's targets in the DCG set, and as such to evaluate which regulators are more likely to be subject-relevant or even causal. Suppose we sift K DCGs from expression profile which contains N genes (there, K and N must have available expression data and were covered by TF2target library). If TF_i has T_i targets in regulation knowledge, there should be $T_i * K/N$ DCGs appeared in TF_i targets list randomly. Actually, it is found that T_I DCGs are included in TF_i's targets list. The larger T_I than $T_i * K/N$ is, the more targets of TF_i enriched, the more likely TF_i is a relevant or causative regulator.

Following is TED formula.

$$TED(TF_i) = -log_2 sum_{x=T_I}^{T_i} C_x^{T_i} (\frac{K}{N})^x (1 - \frac{K}{N})^{T_i - x}$$

Taking the simplified scenario of 13 genes and 23 links in Figure 4 as an example, suppose this expression profile (GSE17967, downloaded from GEO) tested 12632 genes, and 1052 DCGs identified after DCEA. If EGR1 has 4 targets in TF-to-target knowledge, EGR1 should have $4 * 1052/12632$ DCG targets by chance, but the real number is 3. So we take TED formula to calculate TED(EGR1)=$-log_2 sum_{x=3}^{4} C_3^4 (\frac{1052}{12632})^x (1 - \frac{1052}{12632})^{4-x}$=14.34351.

TDD, short for 'Targets' DCL Density', uses Clustering Coefficient to quantify the density of DCLs among a regulator's targets, and so to judge the importance of a TF. Suppose that TF_i has n targets, and that there are k DCLs among these targets. A larger k means more DCLs are bridged by the common TF_i. We intuitively assume that, if a TF bridged more TF_bridged_DCL it is of more importance (even if the regulator is not a DCG). Based on this hypothesis, we employ Clustering Coefficient formula to calculate TDD as follow:

$$TDD(TF_i) = ClusteringCoefficient(TF_i) = \frac{k}{\frac{n*(n-1)}{2}}$$

Again, same example like in TED (Figure 4), EGR1 has 3 DCLs among 4 targets, TDD(Egr-1)=2*3/4(4-1)=0.5.

Of note even though no expression data is available for a TF, its TED and TDD could still be calculated only if the expression level of its targets are measured.

RIF method, short for 'Regulator Impact Factor', simultaneously integrates three sources of information: (i) the extent of differential expression; (ii) the abundance of differentially expressed genes, and (iii) differential co-expression between TF and its differentially expressed target genes to assess which TFs are consistently most differentially co-expressed with the highly abundant and highly differentially expressed genes (Reverter and etal.,2010; Hudson and etal.,2009).

$$RIF(TF_i) = \frac{1}{n_{de}} \sum_{j=1}^{j=n_{de}} [(e1_j * r1_{ij})^2 - (e2_j * r2_{ij})^2]$$

where n_{de} means the number of DEGs, $e1$ ($e2$) means the expression value of DEG_j in condition 1 (condition 2), $r1_{ij}$ ($r2_{ij}$) means the correlation of TF_i and DEG_j in condition 1 (condition 2).

To evaluate the statistical significance of scores which derived by our novel TED and TDD methods, we performed a permutation test, in which we randomly constructed the number of TFąŕs targets-sized pseudo targets for each TF, calculated the new TED scores and TDD scores. This target permutation was repeated many times (Repeat times can be decided by user via a parameter, `Permutation_Times`, the default value is 0. If `Permutation_Times` equal to 0, it indicate that there is no permutation.), and a large number of permutation-generation TED scores and TDD scores formed an empirical null distribution respectively. The p-value and FDR of TED or TDD for each TF can then be estimated.

4 Dataset

DCGL_2.0 includes five datasets: `exprs`, `tf`, `tf2target`, `exprs_design` and `intgenelist`. `exprs`, contains 1000 genes and 63 samples, is a sub-dataset from a real microarray data (GSE17967) from GEO (http://www.ncbi.nlm.nih.gov/geo/). `exprs_design`, required by `DRrank`, elucidates the experiment design of the `exprs`. `tf` and `tf2target`, obtained through processing relevant data (TFbsConFactors.txt and TFbsConsSites.txt) from UCSC hg18, contain 215 human Transcription Factors (TFs) and 214607 TF-to-target relationships. First, two files, TFbsConFactors.txt and TFbsConsSites.txt, were downloaded from UCSC hg18 (http://genome.ucsc.edu/). TFbsConsSites gives predicted chromosomal coordinates of TF binding sites(TFBSs) on human, mouse and rat genes, while TFbsConfactors.txt links the internal TF accessions to SWISS-PROT IDs. Then, these SWISS-PROT IDs were further converted to NCBI gene IDs via BioMart (http://www.ebi.ac.uk/biomart/), and NCBIąŕs homologene.data file was used to find the human homologs of mouse and rat TFs, enabling us to compile an enlarged set of human TF-TFBS relationships. After that, we downloaded gene coordinate information (refGene.txt file), which specifies the chromosomal locations of 18620 human genes. The promoter region of each gene [from 1 kb upstream of the transcription start site (TSS) to 0.5 kb downstream of the TSS] was scanned for the TFBSs identified in the above TF-TFBS relationships. If an occurrence of a certain TFBS was found, the corresponding TF was linked with that gene. In this way, we developed a set of TF-to-target regulatory relationships. In addition, we retrieved TF target information from another source, the TRED database (http://rulai.cshl.edu/TRED/), which collects mammalian cis- and trans-regulatory elements, accompanied by experimental evidence. Finally, `tf2target` (TF-to-target) included 214607 binary tuples involving 215 human TFs and 16863 targets (Tu and etal.,2009). `intgenelist` data is a sample set of user-interested genes, and is required by `DRplot` to plot sub-networks.

5 Examples

5.1 Gene filtration

One can filter genes by `expressionBasedfilter` or `varianceBasedfilter`, keep subset.

```
> library(DCGL)
> data(exprs)
> dim(exprs)

[1] 1000   63

> exprs.filter.1 <- expressionBasedfilter(exprs)
> dim(exprs.filter.1)

[1] 500  63

> exprs.filter.2 <- varianceBasedfilter(exprs, 0.05)
> dim(exprs.filter.2)
```

```
[1] 374  63
```

5.2 DCp: Identifying DCGs

```
> library(DCGL)
> data(exprs)
> exprs[1:3, 1:3]

       Sample1  Sample2  Sample3
AACS  5.267744 5.225570 5.202380
FSTL1 8.629291 8.797554 8.353277
ELMO2 6.096321 6.180715 5.824657
```

`exprs` was designed to study gene expression in cirrhotic tissues with (N=16) and without (N=47) HCC. So we firstly divide `exprs` into two parts corresponding to condition 1 (exprs.1) and condition 2 (exprs.2) respectively.

```
> exprs.1 <- exprs[, 1:16]
> exprs.2 <- exprs[, 17:63]
> DCp.res <- DCp(exprs.1, exprs.2,
+     r.method = c("pearson", "spearman")[1],
+     link.method = c("qth", "rth", "percent")[1],
+     cutoff = 0.25,
+     N = 0,
+     N.type = c("pooled", "gene_by_gene")[1],
+     q.method = c("BH", "holm", "hochberg", "hommel", "bonferroni","BY", "fdr")[1])
> DCp.res[1:3, ]

             dC links p.value q.value
AACS  0.2955923   394      NA      NA
FSTL1 0.3255206   584      NA      NA
ELMO2 0.2687325   642      NA      NA

> DCp.res.N <- DCp(exprs.1, exprs.2,
+     r.method = c("pearson", "spearman")[1],
+     link.method = c("qth", "rth", "percent")[1],
+     cutoff = 0.25,
+     N = 100,
+     N.type = c("pooled", "gene_by_gene")[1],
+     q.method = c("BH", "holm", "hochberg", "hommel", "bonferroni", "BY", "fdr")[1])

10 %
20 %
30 %
40 %
50 %
```

```
60 %
70 %
80 %
90 %
100 %

> DCp.res.N[1:3, ]

             dC links p.value   q.value
AACS  0.2955923   394   0.875 0.9988584
FSTL1 0.3255206   584   0.708 0.9985896
ELMO2 0.2687325   642   0.965 0.9989648
```

Link filter methods(`rLinkfilter, percentLinkfilter and qLinkfilter`) are wrapped in DCp with available parameter 'link.method'. Correlation coefficient methods are also given a option by 'r.method'. So is 'q.method' for adjusting p value methods.

Parameter 'N.type' is used for choosing the permutation type. If 'N.type' is set to 'pooled', that means pooling all the *dC* together to form a null distribution and estimate corresponding statistical significance (p-value) against null statistics. If 'N.type' is set to 'gene_by_gene', that means calculating p-value of a gene only against this gene's null distribution of *dC*.

The 'DCp.res' ia a matrix of all genes with 'dC' column, 'link' column (degree in co-expression networks), 'p.value' column and 'q.value' column. If we set N=0, no permutation has been done, and in this case the 'p.value' and 'q.value' are <NA>.

5.3 DCe: Identifying DCGs and DCLs

As shown in the example of DCp, 'link.mehtod', 'r.method' and 'q.method' are parameters for choosing link-filtration method, correlation-calculating method, and q-value calculating method respectively.

```
> DCe.res <- DCe(exprs.1, exprs.2,
+     link.method = c("qth", "rth", "percent")[1],
+     cutoff = 0.25,
+     r.method = c("pearson", "spearman")[1],
+     q.method = c("BH", "holm", "hochberg", "hommel","bonferroni", "BY", "fdr")[1],
+     nbins = 20, p = 0.1, figname = c("LFC.s.jpeg", "LFC.d.jpeg"))
> DCe.res$DCGs[1:3, ]

       All.links DC.links DCL_same DCL_diff DCL_switch            p            q
CXCL13       411      206       93      101         12 8.433654e-90 8.433654e-87
RPS21        718      250       68      125         57 9.130849e-68 4.565425e-65
METTL5       702      224       54      113         57 2.083395e-53 6.944650e-51
```

'DCe.res' contains two components, one is `DCe.res$DCGs` and the other is `DCe.res$DCLs`.

`DCe.res$DCGs` is a matrix which includes seven columns: 'All.links' (degree of genes in whole co-expression network), 'DC.links' (degree of genes after `Linkfilter`), 'DCL_same'

(the count of same signed correlation coefficient of two conditions in 'DC.links'), 'DCL_diff' (the count of different signed correlation coefficient of two conditions in 'DC.links'), 'D-CL_switch' (the count of switched opposites correlation coefficient of two conditions in 'DC.links'), 'p' (p.value) and 'q' (q.value).

```
> DCe.res$DCLs[1:3, ]

                Gene.1 Gene.2        cor.1      cor.2        type  cor.diff
C9orf45,AACS   C9orf45   AACS -0.679430350 -0.1120171 same signed 0.5674132
ABCD4,AACS       ABCD4   AACS -0.046094800 -0.3431368 same signed 0.2970420
KIAA1661,AACS KIAA1661   AACS  0.008438316  0.3069050 same signed 0.2984666
```

DCe.res$DCLs is a matrix which covers links ('Gene.1' and 'Gene.2'), correlation coefficient ('cor.1', 'cor.2' in two conditions), type ('same signed', 'diff signed' or 'switched opposites') and 'cor.diff' (the absolute value of 'cor.1' minus 'cor.2'). If the user need to narrow down DCGs or DCLs, the may consider setting lower 'cutoff' (in 'qth' or 'percent') or higher co-expression correlation coefficient 'cutoff' (in 'rth') or giving a stricter outlier fraction (p-value).

5.4 DCsum: Summarizing DCGs and DCLs

We implemented DCsum to summarize DCGs and DCLs from 'DCp.res' and 'DCe.res'.

```
> DCsum.res <- DCsum(DCp.res, DCe.res,
+     DCpcutoff = 0.25,
+     DCecutoff = 0.25)
> DCsum.res$DCGs[1:3, ]

     DCG        dC All.links.DCp DCp.p DCp.q All.links.DCe DC.links DCL.same
1  A4GNT 0.5308694           356    NA    NA           356       90       41
2 ADAM23 0.5242025           312    NA    NA           312       71       35
3 ADAM29 0.4779226           596    NA    NA           596      102       56
  DCL.diff DCL.switch        DCe.p        DCe.q
1       38         11 2.493160e-15 1.325344e-13
2       25         11 3.347260e-10 9.297944e-09
3       38          8 6.845184e-07 1.037149e-05

> DCsum.res$DCLs[1:3, ]

               Gene.1 Gene.2      cor.1       cor.2        type  cor.diff
ADAM23; GMPPA  ADAM23  GMPPA -0.5719228 -0.02508201 same signed 0.5468408
ADAM23; CEP350 ADAM23 CEP350  0.6860120 -0.22261784 diff signed 0.9086298
ADAM23; SOD2   ADAM23   SOD2  0.5292947 -0.33945089 diff signed 0.8687456
                  DCG
ADAM23; GMPPA  ADAM23
ADAM23; CEP350 ADAM23
ADAM23; SOD2   ADAM23
```

5.5 DRsort: Sorting out DRGs and DRLs

`DRsort` recommends TF-to-target regulation information which was downloaded from UCSC to identify whether DCGs are TFs or not. If a DCG happened to encode a TF, this DCG is considered to be a DRG. Specially for DCLs, `DRsort` sorts out DCLs to two types, TF2target_DCL and TF_bridged_DCL. Both of them are considered to be DRLs.

```
> data(tf2target)
> DRsort.res <- DRsort(DCsum.res$DCGs, DCsum.res$DCLs, tf2target, exprs)
```

```
> DRsort.res$DRGs[1:3, ]
```

```
     DCG                                           Upstream_TFofDCG
1  A4GNT                                                      CDC5L
2 ADAM23 NF-1;STAT1;PAX3;BRIP1;...;CUX1;MRPL36;DAND5;BACH1;ER-alpha
3 ADAM29                                                         NA
  DCGisTF        dC DCp.p All.links.DCe DC.links DCL.same DCL.diff
1   FALSE 0.5308694    NA           356       90       41       38
2   FALSE 0.5242025    NA           312       71       35       25
3   FALSE 0.4779226    NA           596      102       56       38
  DCL.switch
1         11
2         11
3          8
```

```
> DRsort.res$DRLs[1:3, ]
```

```
         pairID                                    common.TF internal.TF
1 ABHD5; CDC25B                            CREB1; deltaCREB        <NA>
2 ABHD5; USP6NL                                 Egr-1; EGR1        <NA>
3   ABR; AGPAT1 FOS; FOSB; JUN; JUNB; JUND; MIF-1; PLAU; SPZ1        <NA>
  Gene.1 Gene.2      cor.1       cor.2               type  cor.diff    DCG
1  ABHD5 CDC25B  0.5788734 -0.30345618 switched opposites 0.8823296 CDC25B
2  ABHD5 USP6NL -0.4089767  0.46839285        diff signed 0.8773695 USP6NL
3    ABR AGPAT1 -0.8306742 -0.05507074        same signed 0.7756035 AGPAT1
```

```
> DRsort.res$DCGs[1:3, ]
```

```
     DCG                            Upstream_TFofDCG
1  A4GNT                                       CDC5L
2 ADAM23   SP1;NF1;Pax-5;CUX1;MRPL36;DAND5;BACH1;ER-alpha
3 ADAM29                                          NA
  DCGisTF        dC DCp.p All.links.DCe DC.links DCL.same DCL.diff
1   FALSE 0.5308694    NA           356       90       41       38
2   FALSE 0.5242025    NA           312       71       35       25
3   FALSE 0.4779226    NA           596      102       56       38
  DCL.switch
```

```
1         11
2         11
3          8
```

```
> DRsort.res$DCLs[1:3, ]

         pairID                                     common.TF internal.TF
1 ABHD5; CDC25B                             CREB1; deltaCREB        <NA>
2 ABHD5; USP6NL                                  Egr-1; EGR1        <NA>
3   ABR; AGPAT1 FOS; FOSB; JUN; JUNB; JUND; MIF-1; PLAU; SPZ1        <NA>
  Gene.1 Gene.2      cor.1       cor.2               type  cor.diff    DCG
1  ABHD5 CDC25B  0.5788734 -0.30345618 switched opposites 0.8823296 CDC25B
2  ABHD5 USP6NL -0.4089767  0.46839285        diff signed 0.8773695 USP6NL
3    ABR AGPAT1 -0.8306742 -0.05507074        same signed 0.7756035 AGPAT1
```

```
> dim(DRsort.res$DRGs)

[1] 207  10

> dim(DRsort.res$DCGs)

[1] 207  10

> dim(DRsort.res$DRLs)

[1] 4317   10

> dim(DRsort.res$DCLs)

[1] 14059    10
```

`DRGs`, `DRLs`, `DCG2TF`, `TF_bridged_DCL`, `DCGs and DCLs`, six components comprise 'DRsort.res'. 'Upstream_TFofDCG' and 'DCGisTF' columns were added to the list of `DRsort.res$DRGs` to display the differential regulation genes and differential regulated genes. 'common.TF' and 'internal.TF' columns were added to the list of `DRsort.res$DRLs` to identify two type of differential regulated links. Lists of `DRsort.res$DCGs` and `DRsort.res$DCLs` contain all the genes and links came out from `DCsum`, and were annotated regulation information whenever available. And more details were displayed in `DRsort.res$DCG2TF` and `DRsort.res$TF_bridged_DCL` for the ease of follow-up investigation.

5.6 DRplot: Visualizing differential co-expression and regulatory relationships

`DRplot` plots TF2target_DCL-centered (Figure 2) and TF_bridged_DCL-centered (Figure 3) networks depending on *igraph*. Sub-network of TF_bridged_DCL-centered is plotted according to predefined gene 'A2M' which tuned in `intgenelist` (Figure 4).

```
> DRplot.res <- DRplot(DRsort.res,
+     type = c("both", "TF2target_DCL", "TF_bridged_DCL")[1],
+     intgenelist = NULL,
+     vsize=5,asize=0.25,lcex=0.3,ewidth=1,
+     figname = c("TF2target_DCL.pdf", "TF_bridged_DCL.pdf"))

The graph of TF2target_DCL.pdf has been completed and saved in your working directory.
The graph of TF_bridged_DCL.pdf has been completed and saved in your working directory.

> data(intgenelist)
> DRplot.res <- DRplot(DRsort.res,
+     type = c("both", "TF2target_DCL", "TF_bridged_DCL")[3],
+     intgenelist = intgenelist,
+     vsize=5,asize=0.25,lcex=0.3,ewidth=1,
+     figname = c("TF2target_DCL.pdf", "TF_bridged_DCL_int.pdf"))

The graph of TF_bridged_DCL_int.pdf has been completed and saved in your working directory.
```

If 'type' is set to 'TF2target_DCL' or 'TF_bridged_DCL', `DRplot` only plots the chosen network. If 'type' is set to 'both', two networks will be plotted. However, total information of DCGs/DCLs and DRGs/DRLs are not always needed. `DRplot` gives 'intgenelist' parameter which represents a group of interested gene symbols for user to delimit a sub-network.

5.7 DRrank: Ranking regulators

`DRrank` implements three approaches to form a potential rank to show which regulators are more relevant to a phenotypic change or biophysical process in these conditions of expression profiles.

```
> data(tf)
> data(tf2target)
> data(exprs_design)
> DRrank.res <- DRrank(exprs, exprs.1, exprs.2, tf, tf2target,
+     exprs_design, p.value=0.05, DRsort.res, Permutation_Times=0)
> DRrank.res[1:3,]

        TF TED_score TED_rank TED_p.value TED_FDR TDD_score TDD_rank
129 NKX2-5  5.514987        1          NA      NA 0.6875000       18
52   FOXD3  5.133101        2          NA      NA 0.5468750       38
58   FOXO1  5.094599        3          NA      NA 0.4926802       60
    TDD_p.value   TDD_FDR RIF_score RIF_rank
129          NA        NA        NA       NA
52           NA        NA        NA       NA
58           NA        NA  2.261597        7
```

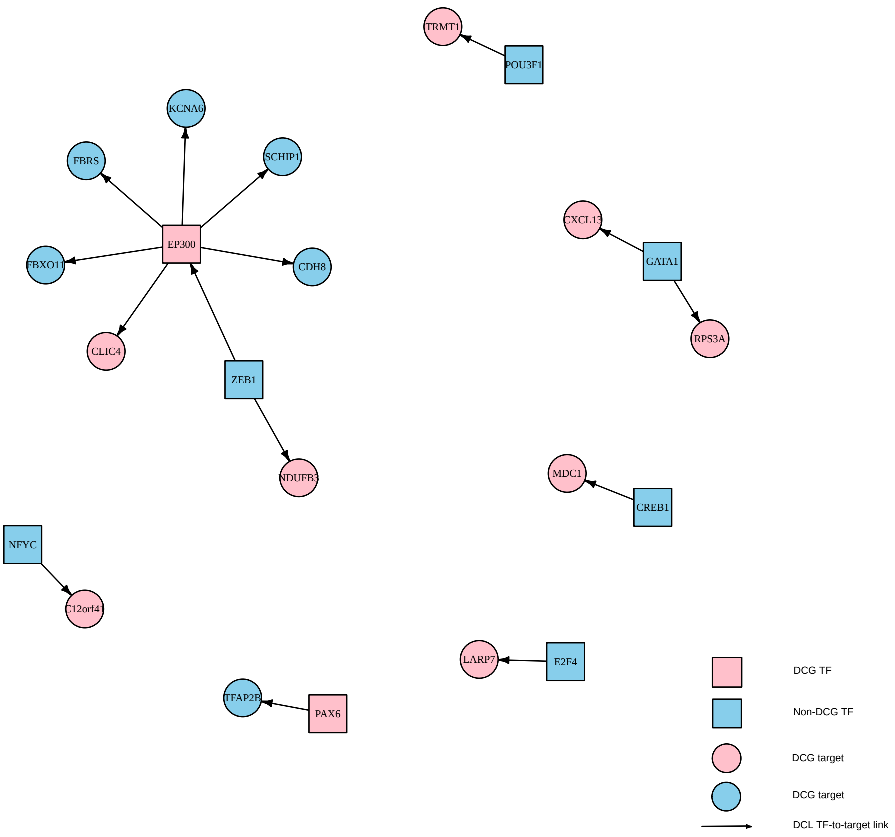

Figure 2: Visualization of TF2target_DCL-centered network. `exprs` was the sample dataset. Nodes represent genes and edges represent DCL TF-to-target link (see symbol illustration).

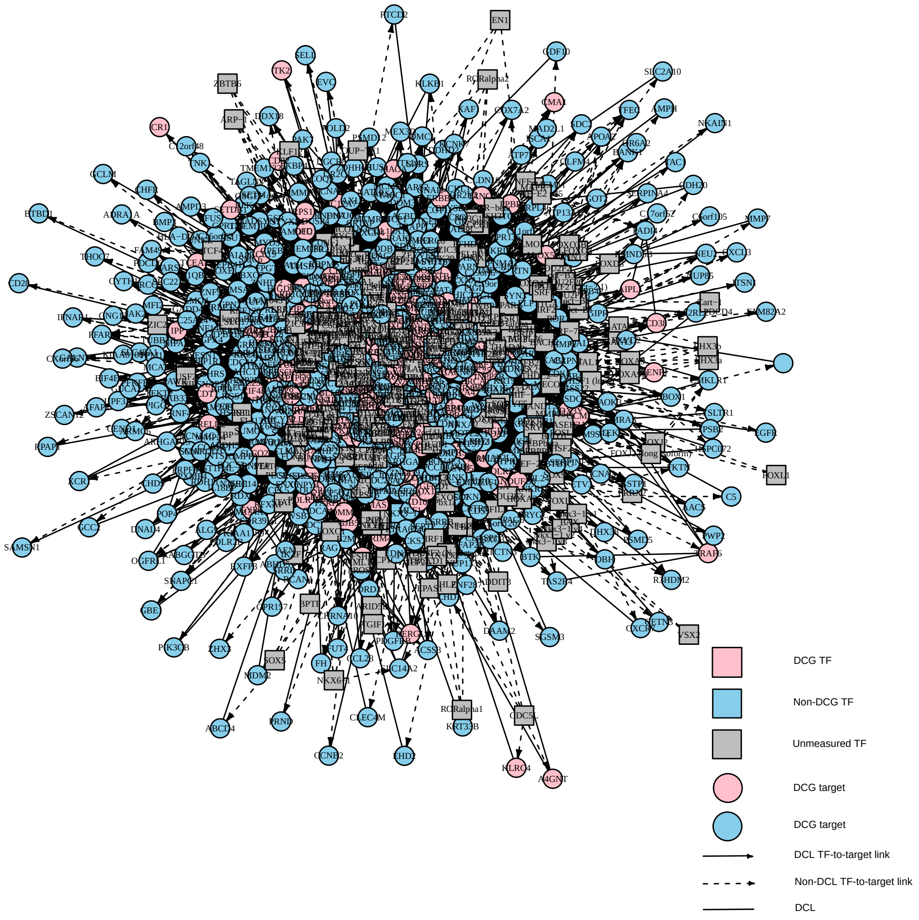

Figure 3: Visualization of TF_bridged_DCL-centered network. `exprs` was the sample dataset. Nodes represent genes and edges represent DCLs or TF-to-target (see symbol illustration).

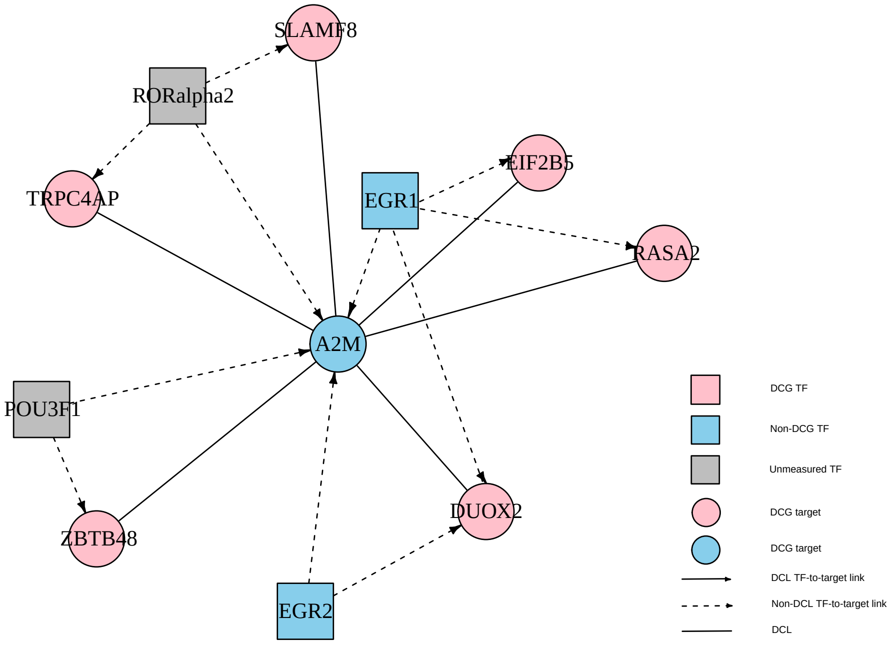

Figure 4: Visualization of TF_bridged_DCL-centered sub-network delimited by predefined gene list. The entire GSE17967 was used as sample dataset, the predefined gene was ‘A2M’. Nodes represent genes and edges represent DCLs or TF-to-target (see symbol illustration).

```
> DRrank.PT.res <- DRrank(exprs, exprs.1, exprs.2, tf, tf2target,
+     exprs_design, p.value=0.05, DRsort.res, Permutation_Times=1000)
10 %
20 %
30 %
40 %
50 %
60 %
70 %
80 %
90 %
100 %
> DRrank.PT.res[1:3,]

        TF TED_score TED_rank TED_p.value TED_FDR TDD_score TDD_rank
129 NKX2-5  5.514987        1  0.00744186    0.48 0.6875000       18
52   FOXD3  5.133101        2  0.01023256    0.48 0.5468750       38
58   FOXO1  5.094599        3  0.01023256    0.48 0.4926802       60
    TDD_p.value   TDD_FDR RIF_score RIF_rank
129  0.04930233 0.5727273        NA       NA
52   0.14418605 0.6792453        NA       NA
58   0.20744186 0.7433333  2.261597        7
```

6 List of abbreviations used

DEA: differential expression analysis
DCEA: differential co-expression analysis
DCG: differentially co-expressed gene
DCL: differentially co-expressed link
DRA: differential regulation analysis
DRG: differentially regulated gene
DRL: differentially regulated link
LRC: Log Ratio of Connectivity
ASC: Average Specific Connectivity
WGCNA: Weighted Gene Co-expression Network
DCp: Differential Co-expression profile
DCe: Differential Co-expression enrichment
GSCA: Gene Set Co-expression Analysis
RIF: Regulatory Impact Factor
TED: Targets Enrichment Density
TDD: Targetsąŕ DCL Density

References

[Reverter and etal.,2010] Reverter, A., Hudson, N.J., Nagaraj, S.H., Perez-Enciso, M. and Dalrymple, B.P. (2010) Regulatory impact factors: unraveling the transcriptional regulation of complex traits from expression data. *Bioinformatics*,26, 896-904.

[Prieto and etal.,2008] Prieto, C., Risueno, A., Fontanillo, C. and De las Rivas, J. (2008) Human gene coexpression landscape: confident network derived from tissue transcriptomic profiles. *PLoS One*,3, e3911.

[Simon and Lam,2006] Simon, R. and Lam, A. (2006) BRB Array Tools Users Guide. Technical Reports. *Biometric Research Branch, National Cancer Institute*

`http://linus.nci.nih.gov/~brb/download_full_new.html`

[Mutch and etal.,2002] Mutch, D. M.,Berger, A.,Mansourian, R.,Rytz, A.,Roberts, M. A. (2002) The limit fold change model: a practical approach for selecting differentially expressed genes from microarray data. *BMC Bioinformatics*, 3, 17.

[Fuller and etal.,2007] Fuller, T.F., Ghazalpour, A., Aten, J.E., Drake, T.A., Lusis, A.J. and Horvath, S.(2007) Weighted gene coexpression network analysis strategies applied to mouse weight. *Mamm Genome*,18, 463-472.

[van Nas and etal.,2009] van Nas, A., Guhathakurta, D., Wang, S.S., Yehya, N., Horvath, S., Zhang, B., Ingram-Drake, L., Chaudhuri, G., Schadt, E.E., Drake, T.A., Arnold, A.P. and Lusis, A.J. (2009) Elucidating the role of gonadal hormones in sexually dimorphic gene coexpression networks. *Endocrinology*,150, 1235-1249.

[Choi and etal.,2005] Choi, J.K., Yu, U., Yoo, O.J. and Kim, S. (2005) Differential coexpression analysis using microarray data and its application to human cancer. *Bioinformatics*, 21, 4348-4355.

[Reverter and etal.,2005] Reverter, A., Ingham, A., Lehnert, S.A., Tan, S.H., Wang, Y., Ratnakumar, A. and Dalrymple, B.P. (2006) Simultaneous identification of differential gene expression and connectivity in inflammation, adipogenesis and cancer. *Bioinformatics*, 22, 2396-2404.

[Yu and etal.,2011] Yu, H., Liu, B.H., Ye, Z.Q., Li, C., Li, Y.X., Li, Y.Y. (2011) Link-based quantitative methods to identify differentially coexpressed genes and gene pairs. *BMC Bioinformatics*, 12, 315

[Liu and etal.,2010] Liu, B.H., Yu, H., Tu, K., Li, C., Li, Y.X., Li, Y.Y. (2010) DCGL: an R package for identifying differentially coexpressed genes and links from gene expression microarray data. *Bioinformatics*, 26, 2637-8

[Reverter and etal.,2010] Reverter, A., Hudson, N. J., Nagaraj, S. H., Perez-Enciso, M., Dalrymple, B. P., (2010) Regulatory impact factors: unraveling the transcriptional regulation of complex traits from expression data *Bioinformatics*, 26, 896-904

[Csardi and etal.,2006] Gabor, C., Tamas, N. (2006) The igraph software package for complex network research *InterJournal*, Complex Systems, 1695

[Elo and etal.,2007] L. L., Elo, H. Jarvenpaa, M., Oresic, R.Lahesmaa and T. Aittokallio. (2007) Systematic construction of gene coexpression networks with applications to human T helper cell differentiation process *Bioinformatics*, 23(16), 2096-103

[Hudson and etal.,2009] Hudson, N.J., A. Reverter, and B.P. Dalrymple. (2009) A differential wiring analysis of expression data correctly identifies the gene containing the causal mutation *PLoS Comput Biol*, 5(5), p. e1000382

[Tu and etal.,2009] Kang Tu, Hui Yu, You-Jia Hua, Yuan-Yuan Li, Lei Liu, Lu Xie and Yi-Xue Li. (2009) Combinatorial network of primary and secondary microRNA-driven regulatory mechanisms *Nucleic Acids Research*, 37(18), 5969ÍC59805
